# Metabolic Variations among Three New Tea Varieties Cultivated in Shandong, China

**DOI:** 10.3390/foods12061299

**Published:** 2023-03-18

**Authors:** Jiazhi Shen, Hui Wang, Litao Sun, Kai Fan, Xifa Zhang, Qingfu Huang, Shibo Ding, Yu Wang, Zhaotang Ding

**Affiliations:** 1 Tea Research Institute, Shandong Academy of Agricultural Sciences, Jinan 250100, China; 2 Tea Research Institute, Rizhao Academy of Agricultural Sciences, Rizhao 276800, China; 3 Tea Research Institute, Qingdao Agricultural University, Qingdao 266109, China; 4 Fuyuanchun Ecological Tea Garden in Wulian County, Wulian, Rizhao 262300, China

**Keywords:** tea varieties evaluation, flavonoids, phenolic acids, amino acids

## Abstract

Cultivar identification is a necessary step in tea breeding programs. Rapid identification methods would greatly improve these breeding processes. To preliminarily identify the three new Lucha tea varieties (LC6, LC7, and LC17) cultivated in Shandong, we measured their main agronomic characters and biochemical components. Then, we analyzed the metabolic profiles of these tea varieties and Fuding Dabaicha (FD) using a UPLC-ESI-MS/MS system. Their biochemical components indicated that the Lucha varieties had excellent varietal characteristics, with higher amino acid contents. Furthermore, secondary metabolism changed a lot in the Lucha tea varieties compared with that in the FD, with their accumulations of flavonoids and phenolic acids showing significant differences. These differential flavonoids were dominated by flavones and flavanone, flavonols, flavonoid carbonosides, and flavanols monomer. Flavanols especially, including epicatechin glucoside, epicatechin-3-(3″-O-methyl)gallate, epigallocatechin-3-O-(3,5-O-dimethyl)gallate, and epitheaflavic acid-3-O-Gallate, showed higher levels in the Lucha varieties. The phenolic acids containing caffeoyl groups showed higher levels in the Lucha varieties than those in the FD, while those containing galloyl groups showed a reverse pattern. Nitrogen metabolism, including amino acids, also showed obvious differences between the Lucha varieties and FD. The differential amino acids were mainly higher in the Lucha varieties, including 5-L-glutamyl-L-amino acid, N-monomethyl-L-arginine, and N-α-acetyl-L-ornithine. By using these approaches, we found that LC6, LC7, and LC17 were excellent varieties with a high yield and high quality for making green teas in Shandong.

## 1. Introduction

The identification and evaluation of tea germplasm resources are the basis of selecting and breeding excellent tea varieties [1,2]. The traditional identification and evaluation of germplasm resources include the use of morphology, biochemistry, molecular markers, and sensory evaluation [3,4,5,6]. Phenotypes can provide a good reference standard for the evaluation of these tea germplasm resources. For instance, the yield of tea depends on the size of the leaf, the density and weight of the bud, and the germination period, while the quality of tea mainly depends on the biochemical composition of the tea bud [7]. A biochemical components analysis can help to understand how the quality characteristics of tea varieties are formed [8]. In recent years, molecular markers have proved to be one of the most effective methods to identify tea varieties [9,10,11].

Although genetics and genomics are greatly contributing to the understanding of the systematic biology of agronomic traits, plant breeding approaches in recent years have considered important information provided by multidimensional data, such as epigenomes, genomes, transcriptomes, proteomes, and metabolomes, which jointly influence phenotypes [12]. These phenotypes are influenced not only by genotypes and the environment, but also by the interactions between the two [13]. The genome affects the phenotype via a series of downstream events, including transcription, translation, and metabolism. Hence, the plant metabolome constitutes a bridge, linking the genotype to the phenotype [12]. Moreover, metabolic information can better reflect the physiological state of plants compared with the proteome and transcriptome [14]. However, the application of metabolomics for trait selection or cultivar identification is still underestimated in tea plant breeding. 

The approach of metabolomics has been applied to expand the current knowledge about the important links between the metabolome and the phenotype in many plants; hence, this facilitates the characterization of their cultivars [15,16,17]. There are also studies that have attempted to reveal the flavor-related information of specific tea cultivars by applying metabolomics [18,19]. Some papers have tried to investigate the relationship between the biochemical components and growth conditions of tea plants via this metabolomics approach [20,21]. However, there are still few reports on the use of metabolomics methods to evaluate and identify tea variety resources. 

Over several years, we found three Lucha varieties, Lucha 6 (LC6), Lucha 7 (LC7), and Lucha 17 (LC17), grown in the Fuyuanchun Ecological Tea Garden in Wulian County, Rizhao, Shandong, China, which showed strong cold and drought resistance properties. To further evaluate the yield and quality of these three varieties for tea breeding, we performed systematic analyses to determine their agronomic characteristics, biochemical components, and metabolomes. In particular, we used metabolomics to systematically analyze the metabolite expression profiles of the three varieties, and identified their differential metabolites compared with the FD in different seasons. This study will pave the way for the examination and registration of the three new Lucha varieties, and their promotion.

## 2. Materials and Methods

### 2.1. Plant Materials

The tea varieties LC6, LC7, LC17, and FD (used as reference), all aged 3 years, were planted in the Fuyuanchun Ecological Tea Garden in Wulian County, Rizhao, Shandong province of China (35°39′ N, 119°27′ E, 41.58 m above sea level). The young shoots (a bud with two leaves) of each variety were plucked randomly on 2 May, 17 June, and 13 October 2021. The samples were marked as LC6M, LC7M, LC17M, FDM, LC6J, LC7J, LC17J, FDJ, LC6O, LC7O, LC17O, and FDO, respectively. These samples were immediately frozen in liquid nitrogen and stored at -80 °C for the metabolome analysis. The lyophilized samples that were used for the metabolome analysis contained three biological replications, as reported by previous research [22,23,24]. In addition, the shoots of the four varieties were also picked to produce green teas. Then, a sensory quality evaluation was performed and the biochemical indices of these green teas were also analyzed. 

### 2.2. Observation and Determination of Morphological Characteristics 

The main morphological characteristics of the four tea varieties were recorded, which included the sprouting time, sprout densities (number of shoots/0.1 m^2^), leaf shape (the ratio of leaf length to leaf width), leaf size (leaf length × leaf width × 0.7, cm^2^), mature leaf color, the pubescence density of a young shoot, and the weight of 100 shoots (g) [7]. The soil and plant analyzer development (SPAD) values and nitrogen contents (N contents) of the four cultivars were determined using a plant nutrient meter (TYS-4N, Topo Yunnong Technology Co., Ltd., Zhejiang, China). The tea leaves were clamped (avoiding veins) using the TYS-4N portable instrument and the number on the screen was read. A total of thirty mature leaves (the third leaf from the top) were randomly selected to detect the SPAD values and nitrogen contents of each cultivar.

### 2.3. Determinations of the Tea Quality Components

The aqueous extract (the water soluble substances in the tea that were leached with boiling water under specified conditions, State Standard of China GB/T 8305-2013), total tea polyphenols, total free amino acids, and caffeine were measured, in accordance with a previous report [25,26]. Each experiment was carried out with at least three replications. The details were as follows.

The grounded tea samples of 3 g (accurate to 0.001g) were transferred to a 500 mL conical flask and then 300 mL of boiling water was added. Then, the conical flask was immediately transferred to a boiling water bath for extraction for 45 min (shake every 10 min). After this extraction, the mixture was immediately vacuum filtered while hot. The tea infusion was stored for biochemical analysis. The tea residue was washed several times with about 150 mL of boiling water, and the tea residue and filter paper of a known quality were moved into the drying dish, where they were then moved together into the constant temperature drying oven. The oven was set 120 °C ± 2 °C. The lid was opened and tilted to the side of the dish. After 1 h, the dish was covered, removed, and cooled for 1 h. Then, the dish was re-transferred to the oven for 1 h. Finally, the dish was immediately moved into the dryer, cooled to room temperature, and weighed. The aqueous extract content in the tea is expressed as a dry mass fraction (%), and calculated according to Equation (1).

(1) $$\mathrm{Aqueous}\;\mathrm{extract}\;\mathrm{content}=\left(1-\frac{m1}{m0\times w}\right)\times 100\%$$

*m*0—the weight of the tea sample, g;
*m*1—the weight of the dried tea residue, g;
*w*—the dry matter content of the tea sample (mass fraction), %.

In total, one milliliter of the stored tea infusion was taken into a 25 mL volumetric flask, where 4 mL of distilled water and 5 mL reaction solution [0.1% FeSO4 and 0.5% potassium sodium tartrate (C_14_H_4_O_6_KNa·4H_2_O)] were added, and the volume was fixed to 25 mL by phosphate buffer (1/15 M Na_2_HPO4, 1/15 M KH_2_PO_4_, pH 7.5). The absorbance was read at 540 nm, using a 10 mm color comparison cell with blank reagents as the controls in a 1810 ultraviolet spectrophotometer (Yoke, Shanghai, China). The tea’s polyphenols content was calculated using Equation (2).

(2) $$\mathrm{Tea}\;\mathrm{polyphenols}\;(\%,\;\mathrm{on}\;a\;\mathrm{dry}\;\mathrm{weight}\;\mathrm{basis})=\frac{A\times 3.914}{1000}\times \frac{L1\times 100}{L2Mm}$$

*L*1—the total volume of the tea infusion, mL;
*L*2—the volume of the infusion taken to reaction, mL;
*M*—the dry weight of the tea sample, g;
*m*—the dry ratio of the tea sample, %;
3.914—corresponded that 1 A using the 10 mm color comparison cell was equal to 3.914 mg of the tea polyphenols in the tea infusion.

A total of one milliliter of the stock tea infusion was taken into a 25 mL volumetric flask. Then, 0.5 mL phosphate buffer (1/15 M Na_2_HPO_4_, 1/15 MKH_2_PO_4_, pH 8.0) and 0.5 mL of 2% ninhydrin (C_9_H_4_O_3_·H_2_O) solution were added to the flask, and it was boiled for 15 min. After fully cooling down, the volume was fixed to 25 mL with distilled water. The OD_570_ was measured using a 5 mm color comparison cell, with blank reagents as the controls, in a 1810 ultraviolet spectrophotometer (Yoke, Shanghai, China). The amino acids content was calculated using Equation (3).

(3) Amino acids (%, on a dry weight basis)=CV11000V2Mw×100

*C*—the amino acids weight (mg), which could be obtained according to the OD_570_ from a standard curve made by theanine or glutamic acid as a standard component, using the same method as mentioned above;
*V*1—the total volume of the tea infusion, mL;
*V*2—the volume of the infusion taken to reaction, mL;
*M*—the dry weight of the tea sample, g;
*w*—the dry ratio of the tea sample, %.

In total, ten milliliters of the stock tea infusion were taken into a 100 mL volumetric flask. Next, 4 mL of 0.01 M HCl and 1 mL of 20% lead acetate basic solution [Pb(CH_3_COO)_2_·Pb(OH)_2_] were added to the flask, then the volume was fixed to 100 mL using distilled water. The reaction was mixed well and kept still on a table for 20 min, and was then filtrated using filter papers. Following this, 25mL of the filtered solution was taken into a 50 mL flask. A total of 0.1 mL of 4.5 M H_2_SO_4_ was added and the volume was fixed to 50 mL with distilled water. After being still on a table for 20 min, the reaction was filtrated again. The OD_274_ was measured using a 10 mm color comparison cell, with blank reagents as the controls, in a 1810 ultraviolet spectrophotometer (Yoke, Shanghai, China). The caffeine content was calculated using Equation (4).

(4) $$\mathrm{Caffeine}\;(\%,\;\mathrm{on}\;a\;\mathrm{dry}\;\mathrm{weight}\;\mathrm{basis})=\frac{\frac{C2.L3}{1000}\times \frac{100}{10}\times \frac{50}{25}}{M2\times m2}\times 100$$

*C*2—the caffeine content (caffeine mg/mL), which could be obtained according to the OD_274_ from a caffeine standard curve made by caffeine as standard component, using the same method as mentioned above;
*L*3—the total volume of the tea infusion, mL;
*M*2—the dry weight of the tea sample, g;
*m*2—the dry ratio of the tea sample, %.

### 2.4. Detection of Metabolites Using UPLC-ESI-MS/MS

The samples that were stored at −80 °C were extracted, as according to a previous study [27]. The details were as follows: the tea samples were freeze-dried by a vacuum freeze-dryer (Scientz-100F). Then, the freeze-dried samples were crushed using a mixer mill (MM 400, Retsch) with a zirconia bead for 1.5 min at 30 Hz. After that, 100 mg of powder was weighed and extracted overnight at 4 °C, with 1.2 mL of a 70% methanol solution. The mixtures were centrifugated at 12,000 rpm for 10 min. The obtained extracts were finally passed through a nylon syringe filter (SCAA-104, 0.22 μm pore size; ANPEL, Shanghai, China, http://www.anpel.com.cn/ (accessed on 12 September 2021)) for a UPLC-MS/MS analysis. A quality control (QC) sample was established to observe the repeatability within an analytical batch.

The obtained sample extracts were analyzed using a UPLC-ESI-MS/MS system (UPLC, Nexera X2, SHIMADZU, Japan; MS, 4500 Q TRAP, Applied Biosystems, Foster City, CA USA). An Agilent SB-C18 (1.8 µm, 2.1 mm × 100 mm) UPLC column was used for separation. The mobile phase consisted of solvent A (pure water with 0.1% formic acid) and solvent B (acetonitrile with 0.1% formic acid). The flow velocity was set as 0.35 mL per minute. The column oven was set to 40 °C. The injection volume was 4 μL. Sample measurements were performed with a gradient program that employed starting conditions of 95% A and 5% B. Within 9 min, a linear gradient to 5% A and 95% B was programmed, and a composition of 5% A and 95% B was kept for 1 min. Subsequently, a composition of 95% A and 5% B was adjusted within 1.1 min and kept for 2.9 min. The effluent was alternatively connected to an ESI-triple quadrupole-linear ion trap (QTRAP)-MS.

Linear ion trap (LIT) and triple quadrupole (QQQ) scans were acquired on a triple quadrupole-linear ion trap mass spectrometer (Q TRAP), AB4500 Q TRAP UPLC/MS/MS System, which was equipped with an ESI Turbo Ion-Spray interface that operated in positive and negative ion mode, and was controlled by Analyst 1.6.3 software (AB Sciex, Framingham, MAUSA). The ESI source operation parameters were set as follows: the ion source was the turbo spray; the source temperature was maintained at 550 °C; the ion spray (IS) voltage was 5500 V in the positive ion mode and 4500 V in the negative ion mode; the ion source gas I (GSI), gas II (GSII), and curtain gas (CUR) were set at 50, 60, and 25.0 psi, respectively; and the collision-activated dissociation (CAD) was high. The instrument tuning and mass calibration were performed with 10 and 100 μmol/L polypropylene glycol solutions in the QQQ and LIT modes, respectively. The QQQ scans were acquired as multiple reaction monitoring (MRM) experiments with the collision gas (nitrogen) set to medium. DP and CE for the individual MRM transitions were performed, with further DP and CE optimization [27]. A specific set of MRM transitions were monitored for each period, according to the metabolites that were eluted within this period.

The identification/annotation of the metabolites that were detected by the UPLC-ESI-MS/MS system was carried out based on a search of the accurate masses of the significant peak features against the online MWDB (metware database from Metware Biotechnology Co., Ltd., Wuhan, China) [27], MassBank (http://www.massbank.jp/ (accessed on 18 September 2021)), KNAPSAcK (http://kanaya.naist.jp/KNApSAcK/ (accessed on 18 September 2021)), HMDB (http://www.hmdb.ca/ (accessed on 19 September 2021)) [28], MoTo Database (http://www.ab.wur.nl/moto/) (accessed on 19 September 2021), and METLIN (http://metlin.scripps.edu/index.php (accessed on 20 September 2021)) [29] databases. The MWDB was based on the MS2T library, which was constructed by Metware Biotechnology Co., Ltd. The annotation of the metabolites in the MS2T library was carried out by matching the fragmentation pattern (delivered by ESI-Q TRAP-MS/MS), the retention time, and the accurate m/z value (delivered by ESI-QqTOF-MS/MS). The metabolites were qualitatively determined according to the secondary spectral information. The isotopic signals, the repeated signals of the K^+^ ions, Na^+^ ions, and NH4^+^ ions, and the repeated signals of the fragment ions, which were other substances with larger molecular weights, were removed during the analysis.

The MultiaQuant software was used to open the mass spectrometry file of the tea samples, and the chromatographic peak integration and calibration were carried out. The peak area of each chromatographic peak represented the relative content of the corresponding substance. Finally, all the chromatographic peak area integration data were derived and stored for further statistical analyses.

### 2.5. Statistical Analyses

An unsupervised PCA (principal component analysis) and supervised PLS-DA (partial least squares discriminant analysis) were performed to preliminarily understand the overall metabolic differences among each group of the samples, using the statistics function prcomp within R (www.r-project.org (accessed on 12 October 2021)). The data were then unit variance scaled before the unsupervised PCA and supervised PLS-DA were carried out. The significantly differential metabolites (SDMs) between the two comparison groups were determined by VIP ≥ 1 and |log_2_ fold change| ≥ 1. The VIP values were extracted from the OPLS-DA (orthogonal partial least squares discriminant analysis) results [30]. The OPLS-DA was generated using R package MetaboAnalyst R [31]. The data were log_2_ transformed and mean centered before the OPLS-DA was executed. In order to avoid overfitting, a permutation test (200 permutations) was performed. 

The annotated metabolites were then mapped to the KEGG pathway database (http://www.kegg.jp/kegg/pathway.html (accessed on 19 October 2021)). The pathways with significantly differential mapped metabolites were then fed into a MSEA (metabolite sets enrichment analysis), and their significance was determined by a hypergeometric test’s *p*-values [32].

The statistical analyses were performed using SPSS 20.0 software (SPSS Inc., Chicago, IL, USA). A one-way analysis of variance (ANOVA) and Duncan’s multiple intervals were used to analyze the significant differences between the two cultivars for their morphological characteristics, and the tea quality components and differences were considered to be statistically significant at *p*-values < 0.05. The graphics were created using GraphPad Prism 7 (La Jolla, CA, USA).

### 2.6. Sensory Evaluation of the Tea Samples

A sensory evaluation was carried out on the green tea made by the LC6, LC7, LC17, and FD young shoots at different seasons, according to the State Standard of China (GB/T 23776-2018) and other recommendations in the literature [33,34]. Briefly, three grams of each manufactured green tea were infused with 150 mL of freshly boiled water for 4 min. The sensory evaluation of these teas and their infusions was carried out by a team of five trained panelists, who assigned scores for the shape of the appearance (25%), the infusion color (10%), the aroma (25%), the infusion taste (30%), and the infused leaves (10%) between 1 and 100, with 1 being bad or “extremely disliked” and 100 being good or “extremely liked”. Finally, the total scores of their organoleptic qualities were calculated based on the weight values.

## 3. Results

### 3.1. Agronomic Characteristics of the Lucha Varieties

**Figure 1:**
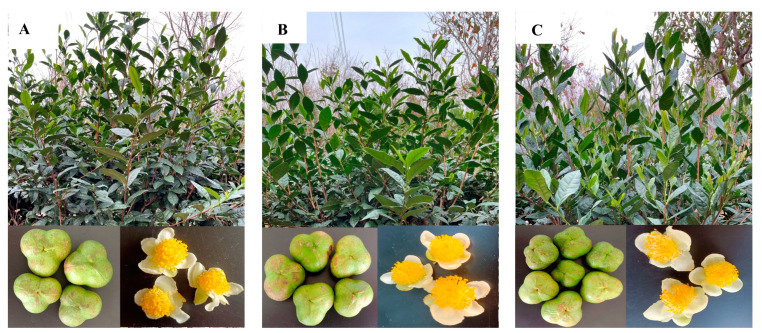
Morphological characters of tea plants, fruits, and flowers of: (**A**) LC6, (**B**) LC7, and (**C**) LC17.

**Figure 2:**
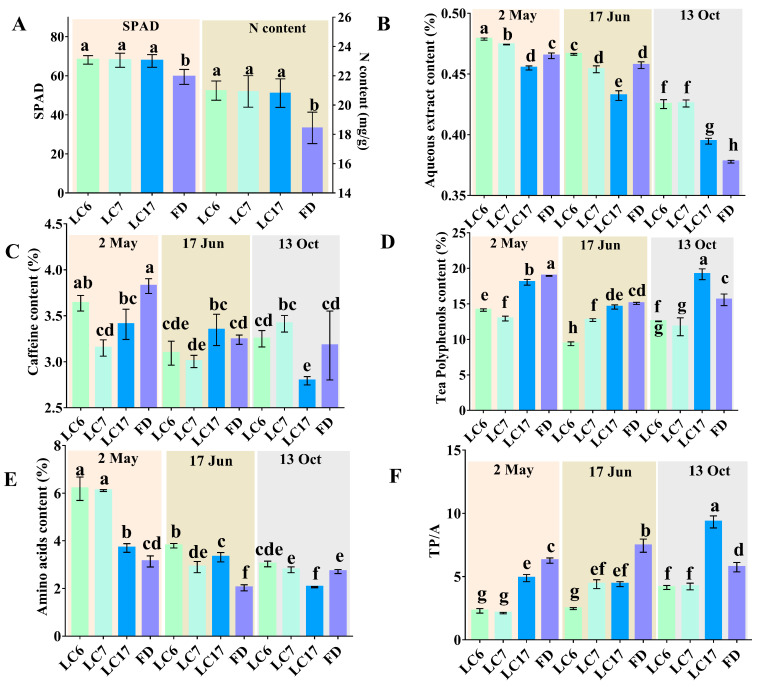
The contents of biochemical compositions in the shoots of the four varieties. (**A**) SPAD values and N contents; (**B**) the aqueous extract contents; (**C**) caffeine contents; (**D**) the tea polyphenol contents; (**E**) the amino acid contents; and (**F**) the ratio of tea polyphenols to amino acids (TP/A ratio). Samples with different lowercase letters differed significantly according to the Duncan test at *p* < 0.05.

To systematically analyze the morphological characters of the three Lucha varieties, their plant shape, leaf type, tea flowers, fruits, and some other agronomic characters were investigated. The results are shown below. The Lucha varieties are shrub types, and their tree postures are all half-expanding types. The shape of the LC7 leaf is a narrow oval, while the shapes of the leaves of the other two varieties are medium ovals. The sizes of the leaves of the three varieties are middle–large. The colors of the mature leaves of the four varieties are dark green (Figure 1). The young buds of all the varieties pose a high density of pubescence. The fruit shapes of LC6 and LC7 are triangular, with three seeds, whereas the fruit shapes of LC17 are triangular or quadrate, with three or four seeds (Figure 1). Furthermore, the yield-related traits of the Lucha varieties were compared with that of the FD. The spouting times of one bud with one leaf are in the following order: FD (15 April), LC6 (18 April), LC7 (21 April), and LC17 (23 April). The densities of the LC6, LC7, LC17, and FD sprouts were about 145, 142, 136, and 123 buds/0.1 m^2^ on 2 May, respectively. The weight of 100 shoots of LC6, LC7, LC17, and FD was, on average, 55 g, 60 g, 65 g, and 55 g, respectively. Likewise, the soil and plant analyzer development (SPAD) value, which represented the relative chlorophyll content, was measured and compared in the cultivars. The SPADs were significantly higher in LC6, LC7, and LC17 than in the FD, and the N contents showed similar trends in the four cultivars (Figure 2A).

### 3.2. Biochemical Components in Different Varieties

To determine the potential of these Lucha varieties to make high-quality teas, their quality-related biochemical components, and those of the FD, were analyzed and compared in three seasons. These biochemical components showed significant differences between the Lucha varieties and the FD, especially in the contents of the tea polyphenols and amino acids. The aqueous extract contents of LC6 and LC7 were higher than that in the FD in the three seasons (Figure 2B). The content of the caffeine in the four varieties varied dramatically according to the seasons. The contents of the caffeine in LC7 and LC17 were significantly lower than that in the FD on 2 May (Figure 2C). To our great interest, the contents of the tea polyphenols in LC6 and LC7 were significantly lower than that in FD at the three time points (Figure 2D). Inversely, the contents of the amino acids in LC6 and LC7 were higher than that in FD, especially on 2 May and 17 June (Figure 2E). The ratios of the tea polyphenols to the amino acids (TP/A ratio) in the tea leaves were also calculated for evaluating the suitability of making teas with LC6, LC7, and LC17. The results showed that the ratios of the polyphenols to amino acids in LC6 and LC7 were significantly lower than that in the FD at the three time points. The ratio in LC17 was also lower than that in the FD, except on 13 October (Figure 2F). The results suggested that the three varieties all had potential values for producing high-quality green teas.

### 3.3. Metabolomic Analysis of UPLC-MS/MS Data

**Figure 3:**
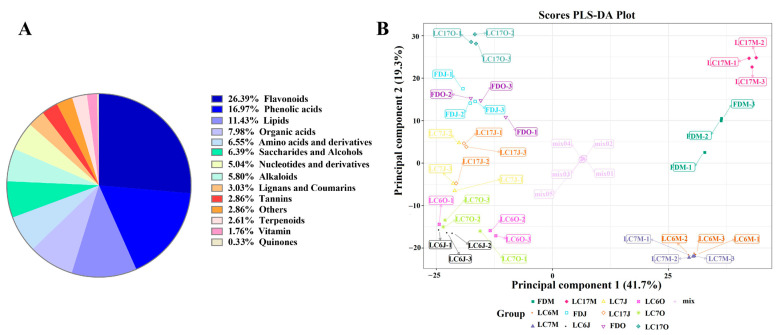
The 1190 metabolites were classified into 14 different chemical classes and different colors of the pie indicate the proportion of different classes of metabolites to total number of metabolites (**A**); and the PLS-DA score plot of leaves from LC6, LC7, LC17, and FD tea varieties at different time points (**B**).

To obtain the metabolites profiling within the tea varieties, the metabolomics were performed using a UPLC-MS/MS analysis. In total, 1190 metabolites were identified in the four tea varieties, according to the databases described in the methods section (Table S1). These metabolites were mainly classified into flavonoids (26.39%), phenolic acids (16.97%), lipids (11.43%), organic acids (7.98%), amino acids and derivatives (6.55%), saccharides and alcohols (6.39%), alkaloids (5.80%), nucleotides and derivatives (5.04%), lignans and coumarins (3.03%), tannins (2.86%), others (2.86%), terpenoids (2.61%), vitamin (1.76%), and quinones (0.34%) (Figure 3A). In this case, the flavonoids and phenolic acids might be typical substances found in tea plants of this tea garden.

To preliminarily understand the overall metabolic differences among each group of the samples, and the variation between the samples within the group, the obtained metabolite profiles were analyzed with an unsupervised principal component analysis (PCA) and a supervised partial least squares discriminant analysis (PLS-DA). In the UPLC-MS/MS-based analysis of the metabolites, the first three principal components of the PCA explained 56.86% of the total variation, and the three varieties were clearly separated from the FD at the three time points (Figure S1). The PLS-DA score plot also showed an obvious separation between the discriminant classes LC6, LC7, LC17, and FD in all three seasons, respectively, with LC6 and LC7 being further from the FD than LC17 was at the three time points. In addition, LC6 and LC7 were closer to each other (Figure 3B). These results demonstrated significant metabolite variations according to the tea varieties. The first two components of the PLS-DA score plot explained 41.7 and 19.3% of the variation, respectively (Figure 3B). The major metabolites that contributed to the first two principal components were epicatechin-3-(3″-O-methyl)gallate, epigallocatechin-3-O-(3,5-O-dimethyl)gallate, kaempferol-3-O-(6″-galloyl)galactoside, kaempferol-3-O-(2″-galloyl)galactoside, and quercetin-3-O-(6″-galloyl)galactoside. 

### 3.4. Metabolomic Variations among the Tea Varieties in Different Seasons

#### 3.4.1. Identification of the Significantly Differential Metabolites

**Figure 4:**
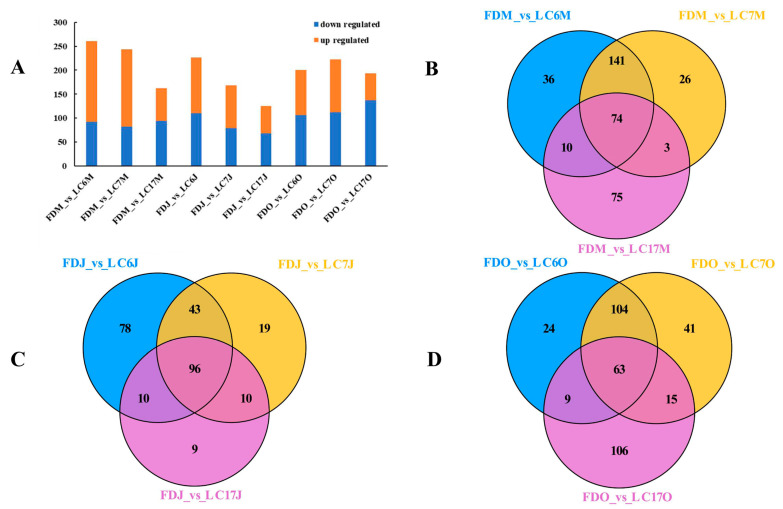
Differential metabolite profiles of LC6, LC7, and LC17 cultivars compared with FD. The number of significantly differential metabolites between Lucha varieties and FD in different seasons. The orange bars meant the number of metabolites with lower levels in Lucha varieties. The grey bars meant the number of metabolites with higher levels in Lucha varieties (**A**). Venn diagram of SDMs in the LC6, LC7, and LC17 cultivars compared with FD in spring (**B**), summer (**C**), and autumn (**D**).

To dissect the variety-dependent metabolic variations, we compared the metabolite profiles of the LC6, LC7, and LC17 varieties with those of the FD, and selected the significantly differential metabolites (SDMs) in the different seasons. The leaf metabolites that passed the VIP ≥ 1 threshold in the OPLS-DA model (*p* < 0.05) and |log_2_fold change| ≥ 1 were considered to be the SDMs between two groups. There were 261, 244, and 162 SDMs in LC6, LC7, and LC17 compared with the FD on 2 May, respectively. Next, 227, 168, and 125 SDMs were found in LC6, LC7, and LC17 compared with the FD on 17 June, respectively. Finally, 200, 223, and 193 SDMs were found in LC6, LC7, and LC17 compared with the FD on 13 October, respectively (Figure 4A). Thus, the number of the SDMs in LC6 and LC7 was more than those in LC17, as compared with the FD.

The Venn diagram showed 36, 26, and 75 unique SDMs in the FDM_vs_LC6M, FDM_vs_LC7M, and FDM_vs_LC17M comparisons in May, respectively. Additionally, 74 SDMs were overlapped in all the comparisons (Figure 4B). In total, 78, 19, and 9 unique SDMs were identified in the FDJ_vs_LC6J, FDJ_vs_LC7J, and FDJ_vs_LC17J comparisons in June, respectively. Additionally, 96 SDMs were overlapped in all the comparisons (Figure 4C). Similarly, 24, 41, and 106 unique SDMs were identified in the FDO_vs_LC6O, FDO_vs_LC7O, and FDO_vs_LC17O comparisons in October, respectively. Furthermore, 63 SDMs were overlapped in all the comparisons (Figure 4D). These results indicated that the metabolite features in the tea plants were also dependent on the seasons. 

#### 3.4.2. K-Means Clustering Analysis of the Significantly Differential Metabolites

**Figure 5:**
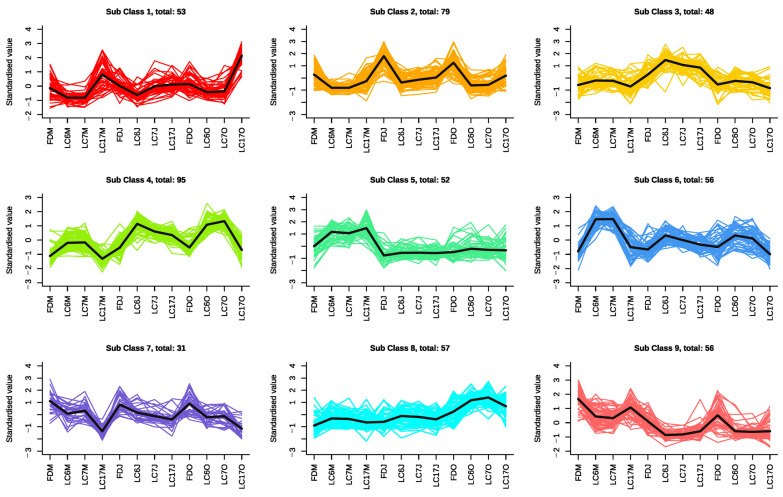
Nine K-means clusters were identified based on relative contents of SDMs in the four cultivars at three time points.

All the SDMs that were identified in our study were clustered based on their relative contents, and were grouped into 9 clusters using the K-means clustering algorithm (Figure 5, Table S2). The SDMs in subclass 1, such as digallic acid, 1,2,6-Tri-O-galloyl-D-glucose, 1,4-Di-O-galloyl-D-glucose, 1,2,3-Tri-O-galloyl-D-glucose, p-dimeric galloyl methyl ester, 1,2,3,6-Tetra-O-galloyl-β-D-glucose, and kaempferol-3-O-galactoside-4′-O-glucoside, showed lower contents in LC6 and LC7 than in the FD and LC17 in all three seasons. The SDMs in subclass 2 presented similar change trends to the SDMs in subclass 1 and presented their highest levels in the FD, including kaempferol-7-O-rhamnoside, kaempferol-3-O-arabinoside, kaempferol-3-O-(6″-galloyl) galactoside, and kaempferol-3-O-(2″-galloyl) galactoside. The SDMs in subclass 3 showed higher contents in LC6 and LC7 than those in the FD and LC17, and these metabolites showed their highest levels in June in all four cultivars. Oxaloacetic acid, glutaric acid, ethylmalonic acid, and apigenin-7-O-(6″-p-Coumaryl) glucoside were included in subclass 3. The SDMs in subclass 4 showed similar change trends to the SDMs in subclass 3, and showed their lowest levels in May in all four cultivars. The SDM contents of subclass 5 were also higher in LC6, LC7, and LC17, and were highest in May in all four cultivars. These metabolites, which included epicatechin-epiafzelechin, epicatechin glucoside, epiafzelechin, and chrysin-5-O-glucoside, showed no variations among the cultivars in the other two seasons. The SDMs of subclass 6 showed higher contents in LC6 and LC7 than those in the FD and LC17, and were highest in May in LC6 and LC7. Metabolites such as dicaffeoylshikimic acid, 3,4,5-tricaffeoylquinic acid, 1-O-Caffeoyl xyloses, 5-L-Glutamyl-L-amino acid, homoarginine, and L-Aspartyl-L-Phenylalanine were included in subclass 6. The contents of the SDMs in subclass 7 descended in order in the FD, LC6, LC7, and LC17 in the three seasons. Subclass 8 contained SDMs which showed higher contents in LC6 and LC7. Meanwhile, these metabolites were highest in the four varieties in October. The contents of the SDMs in subclass 9 decreased with seasonal changes in the four varieties.

#### 3.4.3. Variation in Flavonoids between the Lucha Varieties and FD in Different Seasons

**Figure 6:**
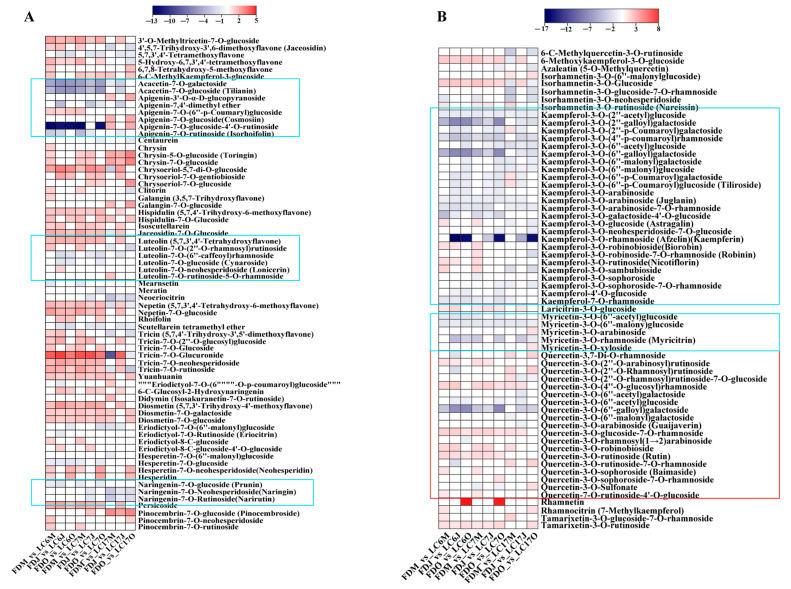

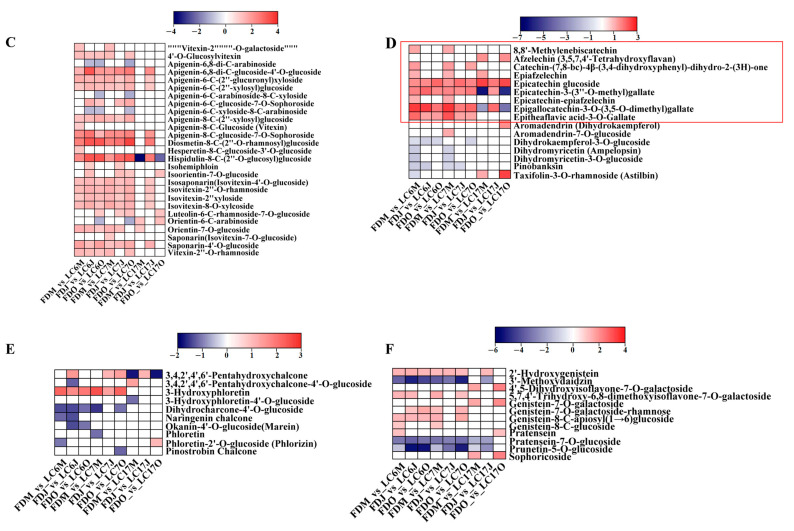
The heatmap is shown to represent the alteration of flavonoids including (**A**) flavones and flavanones, (**B**) flavonols, (**C**) flavonoid carbonosides, (**D**) flavanols, (**E**) chalcones, and (**F**) isoflavones in LC6, 7, and 17 against the FD in May, June, and October, respectively. Data are shown as log_2_ fold change. Significantly upregulated metabolites are shown in red. Significantly downregulated metabolites are shown in blue. Non-significantly regulated metabolites are shown in white.

To access the metabolism characteristics of LC6, LC7, and LC17, the national tea district test standard control species, FD, was used as the control. Based on the OPLS-DA models, the key differential metabolites that contributed to the differentiation of Lucha and FD were identified in the different seasons. A significant variation was observed in the composition and content of the flavonoids between the Lucha varieties and the FD in the different seasons (Figure 6). The flavones and flavanones belonging to the flavonoid class showed higher accumulations mainly in the three Lucha shoots compared with the FD (Figure 6A). However, the metabolites that included apigenin glycosides such as apigenin-7-O-glucoside-4′-O-rutinoside, luteolin glycosides such as luteolin-7-O-(6″-caffeoyl)rhamnoside, and naringenin glycosides such as naringenin-7-O-glucoside and naringenin-7-O-neohesperidoside, showed higher levels in the FD. Quercetin glycosides, a type of flavanols, had higher accumulations in the three Lucha shoots compared with the FD, whereas kaempferol and myricetin glycosides showed lower levels in the Lucha shoots (Figure 6B). Another subclass of flavonoids that presented with significant changes was flavonoid carbonosides (Figure 6C). These metabolites had higher contents in the Lucha varieties compared with the FD. Among them, most of the apigenin carbonosides showed no significance in LC17 compared with the FD, which were different from those in LC6 and LC7. The accumulation of flavanols and flavanonols also changed, to some extent, between the Lucha varieties and the FD. The flavanols, such as epicatechin glucoside, epicatechin-3-(3″-O-methyl)gallate, epigallocatechin-3-O-(3,5-O-dimethyl)gallate, and epitheaflavic acid-3-O-Gallate, accumulated more, while the flavanonols accumulated less in the Lucha varieties compared with the FD (Figure 6D). Moreover, chalcones and isoflavones also presented with significant differences between the Lucha varieties and the FD (Figure 6E,F). These two subclasses showed an opposite pattern of difference. That is, chalcones such as dihydrocharcone-4′-O-glucoside and naringenin chalcone presented with lower levels in the Lucha varieties, while isoflavones such as genistein-7-O-galactoside-rhamnose and genistein-8-C-apiosyl(1→6)glucoside showed higher levels in the Lucha varieties. 

#### 3.4.4. Variation in Phenolic Acids between the Lucha Varieties and FD in Different Seasons

**Figure 7:**
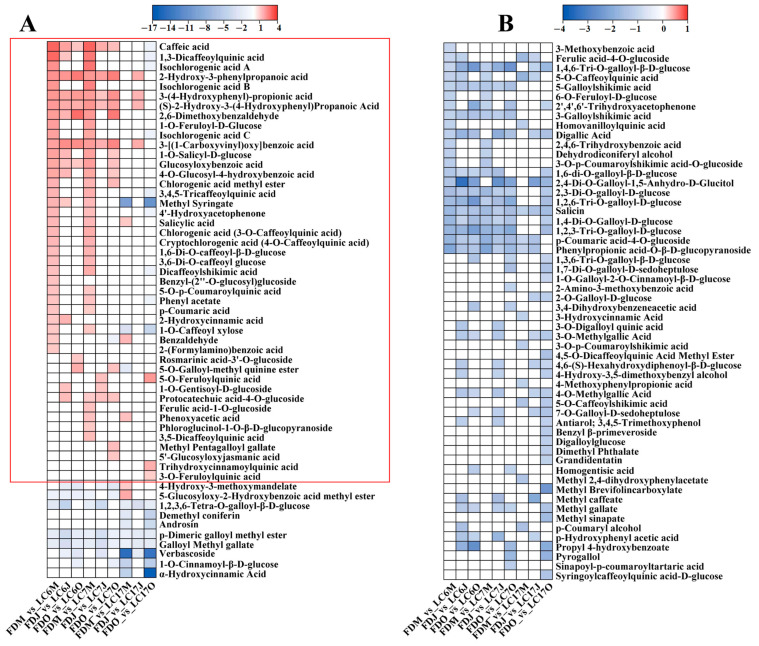
The heatmap is shown to represent the alteration of phenolic acids in LC6, 7, and 17 against the FD in May, June, and October, respectively. (**A**) phenolic acids with larger |log_2_ fold change|, (**B**) phenolic acids with smaller |log_2_ fold change|. Data are shown as log_2_ fold change. Significantly upregulated metabolites are shown in red. Significantly down-regulated metabolites are shown in blue. Non-significantly regulated metabolites are shown in white.

Using the OPLS-DA models, we found that phenolic acid was the second largest class of SDMs that were obtained between the Lucha varieties and the FD (Figure 7A,B). The phenolic acids that were identified in this study were mainly hydroxycinnamic acid- and hydroxybenzoic acid-types, and their derivates. There were 111 phenolic acids that presented with significant differences in the Lucha varieties and FD. The number of phenolic acids which presented with higher levels in the LC6 and LC7 shoots were more than those that presented with higher levels in the FD in May. However, the number of phenolic acids with higher levels in the LC6 and LC7 shoots dramatically decreased in June and October. Additionally, most of the phenolic acids, such as caffeic acid, glucosyloxybenzoic acid, and 4-O-glucosyl-4-hydroxybenzoic acid, with significantly higher levels in LC6 and LC7, showed no significant differences or lower levels in LC17 compared with the FD (Figure 7A). Interestingly, at any of the three points, most of the phenolic acids presented with higher levels in the FD shoots than in LC17. This result was different from the LC6_vs_FD and LC7_vs_FD comparisons in May. The phenolic acids containing caffeoyl groups showed higher levels in the Lucha varieties than in the FD, while those containing galloyl groups showed a reverse pattern, which was an interesting discovery. These results indicated that the secondary metabolism of the Lucha varieties changed, to some extent, compared with the FD.

#### 3.4.5. Variation in Amino Acids and Alkaloids between the Lucha Varieties and FD in Different Seasons 

**Figure 8:**
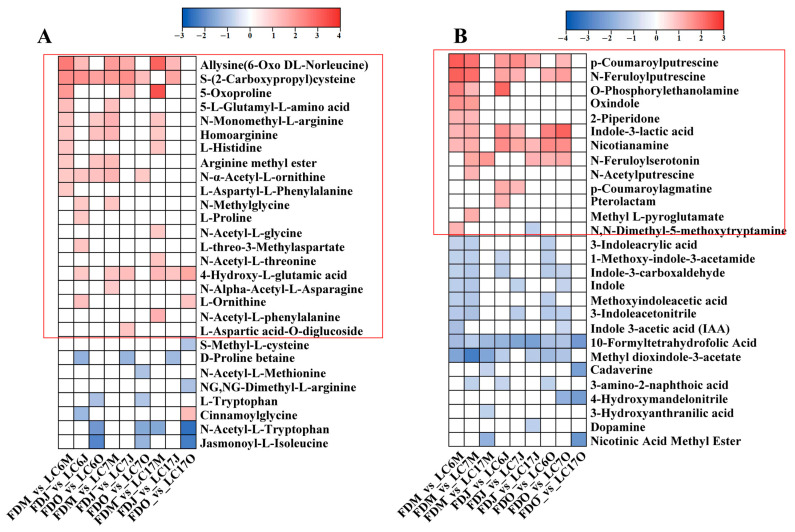
The heatmap is shown to represent the alteration of amino acids (**A**) and alkaloids (**B**) in LC6, 7, and 17 against the FD in May, June, and October, respectively. Data are shown as log_2_ fold change. Significantly upregulated metabolites are shown in red. Significantly down-regulated metabolites are shown in blue. Non-significantly regulated metabolites are shown in white.

Amino acids are attributed to the umami taste, and their content within tea shoots is one of the key determinants for green tea quality. Therefore, it is necessary to analyze the amino acid metabolism in the Lucha varieties. The differential amino acids were mainly amino acid derivates, such as N-acetyl-L-threonine, 4-hydroxy-L-glutamic acid, homoarginine, and N-monomethyl-L-arginine. There were 28 amino acids that presented with significant differences in the Lucha varieties and the FD (Figure 8A). As expected, most of the differential amino acids showed higher levels in the Lucha varieties than in the FD. However, the amino acids with a higher content in the different Lucha varieties were a little different. For example, arginine methyl ester and N-α-acetyl-L-ornithine showed higher levels in LC6 and LC7, while showed a significant difference in LC17. N-Acetyl-L-phenylalanine, N-Acetyl-L-glycine, and N-acetyl-L-threonine only presented significant differences in LC17.

The alkaloid metabolisms that showed a discrepancy between the LC varieties and FD were also found. There were 28 alkaloids presenting with significant differences in the Lucha varieties and the FD (Figure 8B). The differential alkaloids were mainly amine compounds and indole alkaloids, such as N,N-dimethyl-5-methoxytryptamine, O-phosphorylethanolamine, nicotianamine, 3-indoleacrylic acid, 1-methoxy-indole-3-acetamide, indole-3-carboxaldehyde, and indole. The differential alkaloids that accumulated higher levels in the LC varieties were mainly amine compounds, such as p-coumaroylputrescine, N-feruloylputrescine, and O-phosphorylethanolamine, whereas the alkaloids that accumulated higher levels in the FD were indole alkaloids, 3-indoleacrylic acid, 1-methoxy-indole-3-acetamide, indole-3-carboxaldehyde, indole, methoxyindoleacetic acid, and 3-indoleacetonitrile. 

### 3.5. Sensory Evaluation of the Green Teas Made by LC6, LC7, LC17, and FD

To intuitively evaluate the qualities of the green teas that were made from the three Lucha varieties, sensory quality evaluations of the teas that were made in the spring, summer, and autumn were conducted, and their scores of sensory indexes were compared with those of the green teas made from the FD (Table 1). The total scores of the sensory indexes did not show significant differences between the green teas made from the Lucha and FD varieties. However, the score of the infusion taste, which accounts for 30% of the total score, had higher levels in the green teas made from LC6 and LC7, especially in the spring. In this regard, it is considered that the three Lucha varieties are suitable for producing a high quality of green tea with a pleasant taste.

## 4. Discussion

Metabolites are the end products of cellular regulation, and their levels can be viewed as the ultimate responses of a creature to genetic or environmental changes [35]. The qualitative and quantitative changes in primary and secondary metabolites with low molecular weights could directly modulate phenotypes, which would be considered as markers for specific trait selection and variety identification or characterization [36,37]. Therefore, metabolomics has broad application prospects in accelerating the breeding process, and has recently been applied in crop metabolomic-assisted breeding [16,38,39]. In this study, we tried to apply metabolomics to evaluate three new tea varieties (LC6, LC7, and LC17) selected in Shandong. These three varieties had undergone a long-term adversity domesticated in Shandong environmental conditions, and had formed important agronomic traits, such as stronger cold and drought resistance and dark green leaves.

To access the metabolic traits of LC6, LC7, and LC17, the UPLC-MS/MS-based metabolites were detected and a comparative metabolomic study between the Lucha varieties and the FD were carried out in different seasons. The structural classes of the metabolites within these varieties showed diversity, ranging from primary metabolites such as saccharides and alcohols, amino acids and derivatives, and organic acids, to very complex secondary metabolites such as flavonoids, phenolic acids, lipids, alkaloids, nucleotides and derivatives, tannins, terpenoids, vitamins, and quinones (Table S1). Additionally, these metabolites varied over the seasons in each of the four varieties in a specific pattern (Figure 5). The comparative metabolomic results showed that the discriminant metabolite species were dominated by flavonoids, followed by phenolic acids, alkaloids, amino acids, and derivatives, in different seasons. The major differential metabolite categories that were identified here can provide invaluable information on the biochemical mechanisms in the Lucha varieties. For example, the higher levels of flavonols that accumulated in the Lucha varieties were mainly glycoside derivates, whereas those with higher contents in the FD shoots were mainly acyl glycoside derivates. Flavanols including epicatechin glucoside, epicatechin-3-(3″-O-methyl)gallate, epigallocatechin-3-O-(3,5-O-dimethyl)gallate, and epitheaflavic acid-3-O-gallate were only found more accumulated in LC6, LC7, and LC17. The differential accumulation pattern of the catechins and favonol glycosides in the different tea varieties and cultivars had also been reported previously [40]. Another metabolite class, flavonoid carbonosides, also accumulated more in the three Lucha varieties, especially in spring. All these higher-accumulated flavonoid compounds could be considered to be characteristic compounds of the Lucha varieties. Flavonoids are synthesized through the phenylpropanoid pathway, in which cinnamic acid is converted to 4-coumaroyl-CoA. Then, 4-coumaroyl-CoA enters the flavonoid biosynthesis pathway, and the flavone and flavonol biosynthesis pathway [41]. Correspondingly, the KEGG analysis showed that the differential metabolites between the Lucha varieties and the FD were notably enriched in the flavonoid biosynthesis (ko00941), phenylpropanoid biosynthesis (ko00940), and flavone and flavonol biosynthesis (ko00944) pathways (Figure S2). 

Flavonoids have a range of biological activities in plants, such as antioxidant activities, antimicrobial activities, signaling, and defense against environmental stressors [42]. It has been reported that the flavonoids biosynthesis pathway was one of the most affected during the domestication and selection of chili pepper [43]. We suspected that the Lucha varieties strengthened this route to cope with the cold weather or other adverse environments in Shandong. Furthermore, phenolic acids also presented a Lucha-varieties-specific accumulation. Those phenolic acids with a high content in the Lucha varieties were cinnamic acid- and benzoic acid-types and their derivates, while the phenolic acids with a high content in the FD were benzoic acid-type, and were mainly derivatives of gallic acid. Phenolic compounds are also crucial for plant growth and reproduction, and are produced as a response to environmental factors and to defend injured plants [41]. Since many plant metabolites are family or species-specific, they are at least likely to fulfil some important biological functions, such as helping plants to survive some specialized ecological niches [35]. Therefore, the above results indicated that the Lucha varieties reprogrammed their metabolisms to produce more bioactive secondary metabolites, such as flavonoids and phenolic acids, which could be a characteristic adaption mechanism for the survival of the Lucha varieties under Shandong environments. 

These differential metabolites would also give the final tea product that was manufactured from the Lucha varieties unique qualities. Flavan-3-ols were considered to play an essential role in imparting the bitterness and astringency [44] in teas. The differential flavanols that were only higher in the Lucha varieties would contribute to the astringent taste of the tea. Similar results have also been found in a white tea report, in which higher levels of flavan-3-ols exhibited strong correlations with the greater bitterness and astringency taste of white teas [45]. Phenolic compounds were reported to impact the unique taste, flavor, and health-promoting properties in vegetables and fruits [46]. In addition, phenolic acids and their derivatives, for example, theogallin, chlorogenic acid, 4-caffeoylquinic acid, and 3,5-dicaffeoylquinic acid, were found to present positive correlations with bitterness and astringency taste [45]. That is, the acylation of polyphenols contributed to the grassy and astringent taste of the tea samples [44]. In our study, we also detected some acylation phenolic acids, such as 3,4,5-tricaffeoylquinic acid, chlorogenic acid (3-O-caffeoylquinic acid), cryptochlorogenic acid (4-O-caffeoylquinic acid), and dicaffeoylshikimic acid, which were higher in the Lucha varieties. Therefore, increasing the phenolic content in tea plants could enhance the tea quality and the mellow and thick tastes of the tea infusion. The higher score of the infusion taste from the sensory quality evaluations also confirmed this opinion. It is thought that many of the bioactive compounds that presented in the final tea product and were related to its quality or functional properties were formed during the tea manufacturing process [47]. Given that the manufacturing process of green tea does not involve intense oxidation, its quality is believed to be determined by the intrinsic chemical compositions of fresh tea leaves [48,49]. Therefore, the higher accumulated flavonoids in the fresh shoots of the Lucha varieties laid a fine foundation for the quality of the final tea product.

Amino acids are another major component that determine the quality of tea. Although free amino acids account for only 1% to 4% of the dry weight of tea, they are closely associated with the taste and aroma of tea [48]. Our results showed that the Lucha varieties contained higher amino acid contents compared with those in the FD in the three seasons, which might give the Lucha tea its umami taste. Flavor active peptides, amino acids, and amino acid derivatives are the main flavor substances of many fermented foods which have a bitter taste, umami taste, or kokumi taste [50]. To be specific, Gly, Ala, Val, Leu, Tyr, and Phe and their peptides impart bitterness [51]. Pyroglutamic acid, pyroglutamyl-Pro-X peptides, glutamate, and α-Glutamyl di-, and tripeptides, especially Asp-, Thr-, and Ser-containing peptides, impart umami taste [51], while glutathione and several γ-glutamyl dipeptides are kokumi active compounds [52]. In the present study, the differential amino acids, especially 5-oxoproline (pyroglutamic acid) and 5-L-glutamyl-L-amino acid, could contribute to the umami taste of the tea made by the Lucha varieties. We did not observe many bitter or kokumi taste amino acids that presented significantly higher levels in the Lucha varieties than in the FD, even during summer or autumn. The composition and higher contents of the amino acids in the Lucha varieties might also contribute to the higher score of the infusion taste from the sensory quality evaluations. All the obtained results were fine indications that the Lucha varieties were qualified to be high-quality cultivars.

## 5. Conclusions

By using a combination of agronomic characters, biochemical components, and metabolomic analyses, we performed an extensive characterization of three new tea varieties that were cultivated in Shandong in different seasons. By using supervised, predictive learning algorithms (OPLS-DA), we extracted the metabolic features contributing to the differentiation of the Lucha varieties and the FD. Those differential metabolites between the Lucha varieties and the FD mainly included flavonoids, phenolic acids, and amino acids, which give the final tea product unique qualities. Therefore, we believe that our Lucha varieties are high-quality cultivars that are suitable for planting in Shandong. The study will lay the foundation for the examination and registration of the three Lucha new varieties, and their promotion.

## Figures and Tables

**Table 1:** Scores of sensory evaluation of green teas made by different varieties in spring, summer, and autumn.

Sensory Indexes (SIs)	Spring (2 May)				Summer (17 June)				Autumn (13 October)			
(SI%)	LC6M	LC7M	LC17M	FDM	LC6J	LC7J	LC17J	FDJ	LC6O	LC7O	LC17O	FDO
Appearance (25%)	92.83 ± 0.16 a	92.33 ± 0.33 ab	91.5 ± 0.28 abc	92 ± 0.57 ab	90.17 ± 0.16 c	91.83 ± 0.16 ab	92.5 ± 0.28 ab	92.17 ± 0.60 ab	90.5 ± 0.5 cd	92.5 ± 0.28 ab	90.5 ± 0.76 cd	91.17 ± 0.16 bcd
Aroma (25%)	90.5 ± 0.28 de	90.33 ± 0.33 e	90.83 ± 0.44 de	91.33 ± 0.33 cd	93.17 ± 0.16 a	92.17 ± 0.16 bc	91.83 ± 0.16 bc	90.5 ± 0.28 de	90.33 ± 0.33 e	92.33 ± 0.33 ab	92 ± 0 bc	93.17 ± 0.16 a
Infusion color (10%)	90.5 ± 1.04 d	92 ± 0.28 bcd	91 ± 0.57 d	91.17 ± 0.72 cd	91.17 ± 0.16 cd	91 ± 0.28 d	92.83 ± 0.16 abc	91.33 ± 0.72 cd	94 ± 0.57 a	93.5 ± 0.28 ab	90.67 ± 0.33 d	91 ± 0.57 d
Infusion taste (30%)	92.33 ± 0.33 a	92.33 ± 0.33 a	90.67 ± 0.33 bc	91.33 ± 0.33 b	90.33 ± 0.33 cd	89.67 ± 0.33 d	92.33 ± 0.33 a	90.33 ± 0.33 d	92.5 ± 0.28 a	92.5 ± 0.28 a	92.67 ± 0.16 a	92.67 ± 0.16 a
Infused leaf (10%)	92.67 ± 0.33 a	92.33 ± 0.33 a	92.33 ± 0.33 a	91.83 ± 0.44 abc	92 ± 0 ab	91.33 ± 0.33 bcd	90.5 ± 0.28 de	91 ± 0 cde	92.17 ± 0.16 ab	92.5 ± 0.28 a	90.33 ± 0.33 e	90.67 ± 0.33 de
Overall	91.85	91.80	91.12	91.53	91.25	91.13	92.11	91.00	91.57	92.56	91.53	92.05

Note: Total score of sensory indexes (SI) is 100, which includes appearance, aroma, infusion color, infusion taste, and infused leaf color/shape contributing 25%, 25%, 10%, 30%, and 10% of SI, respectively; lowercase letters indicate statistical significance; and samples not sharing a letter differed significantly according to the Duncan test at *p* < 0.05.

## Data Availability

The data used to support the findings of this study are included within the supplementary information files.

## Supplementary Materials

The following supporting information can be downloaded at: https://www.mdpi.com/article/10.3390/foods12061299/s1, Figure S1: The PCA score plot of leaves from LC6, LC7, LC17, and FD tea varieties of different time points; Figure S2: The top 20 of KEGG enrichment of SDMs in young shoots of LC6, LC7, and LC17 in different seasons; Table S1: The detail information of the 1190 identified metabolites; Table S2: The cluster distribution of the differential metabolites according to the K-means clustering analysis.
