# Supplementary material for: Metabolic Variations among Three New Tea Varieties Cultivated in Shandong, China
Source: Foods. 2023 Mar 18;12(6):1299. doi: 10.3390/foods12061299 (PMC10048610; doi:10.3390/foods12061299)
Supplement: Supplementary file 1 [file foods-12-01299-s001.zip › Figure S1 and Figure S2.pdf]

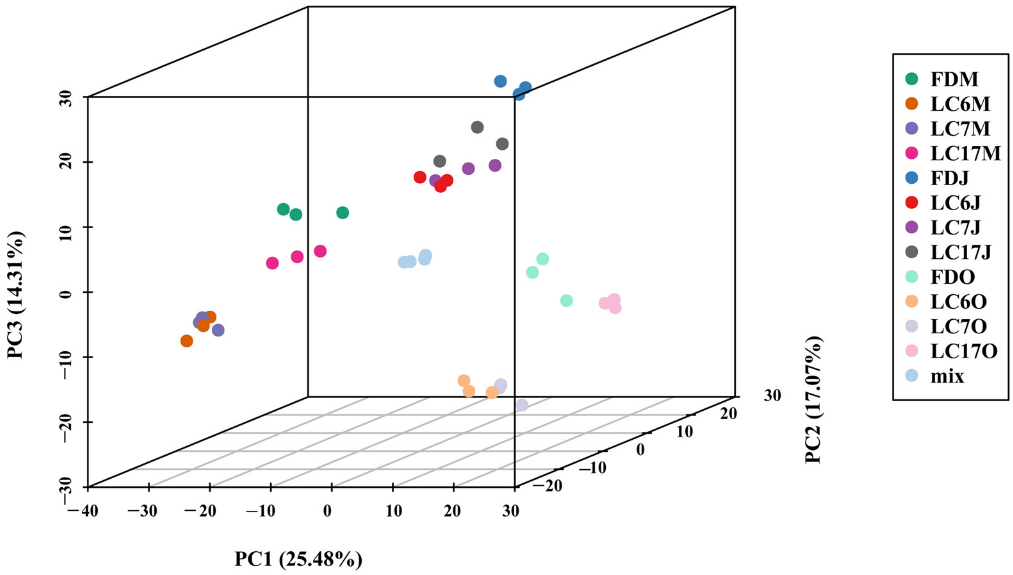

Figure S1. The PCA score plot of leaves from LC6, LC7, LC17 and FD tea varieties of different time points.

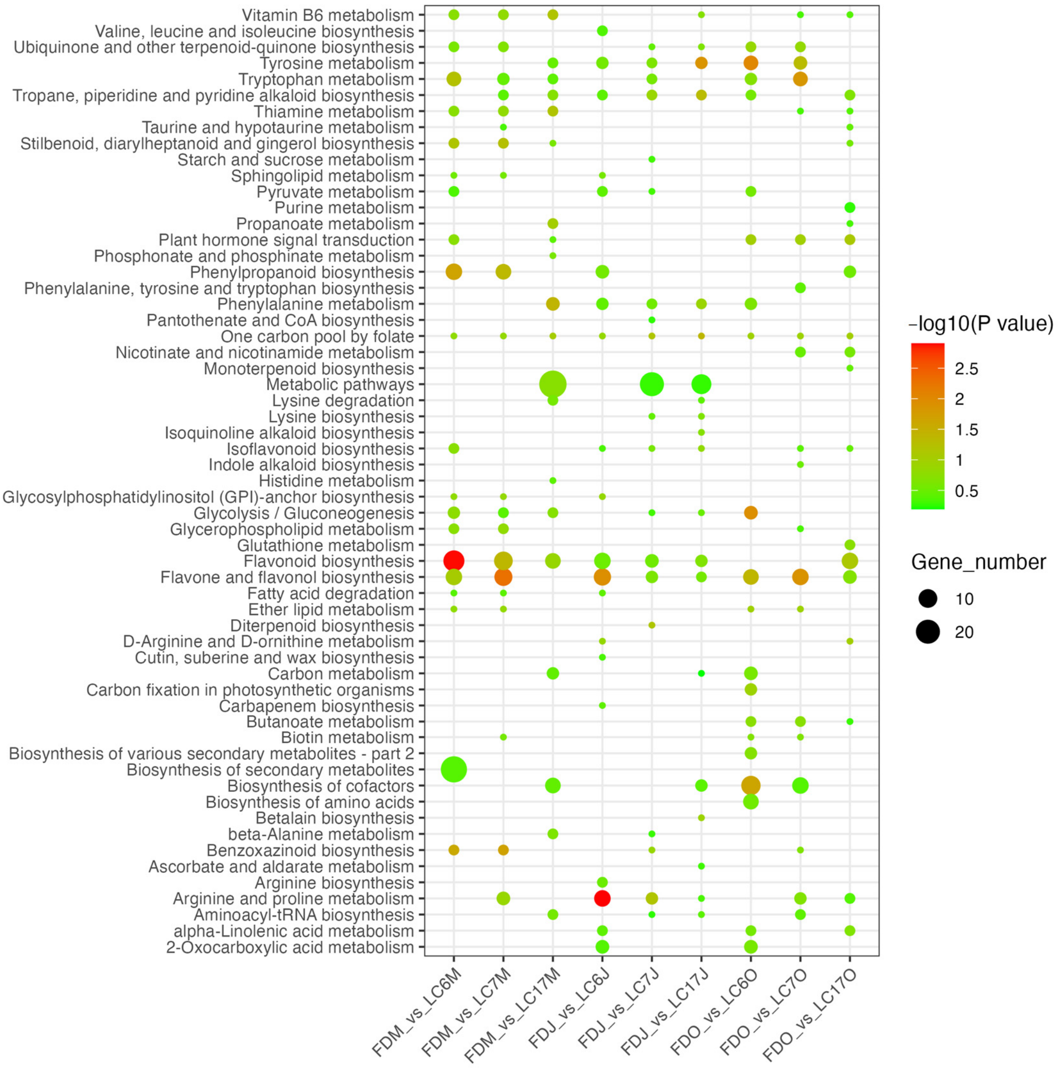

Figure S2. The top 20 of KEGG enrichment of SDMs in young shoots of LC6, LC7 and LC17 in different seasons.
